# MACRONUTRIENTS OF MOTHERS’ MILK OF VERY LOW BIRTH WEIGHT INFANTS: ANALYSIS ACCORDING TO GESTATIONAL AGE AND MATERNAL VARIABLES

**DOI:** 10.1590/1984-0462/2021/39/2019097

**Published:** 2020-06-22

**Authors:** Maria Elisabeth Lopes Moreira, Sabrina Lopes Lucena, Patrícia Sffeir Coelho de Magalhães, Adriana Duarte Rocha, Ana Carolina Carioca Costa, Fernanda Valente Mendes Soares

**Affiliations:** a Instituto Nacional de Saúde da Mulher e da Criança Fernandes Figueira, Rio de Janeiro, RJ, Brazil.

**Keywords:** Macronutrients, Milk, human, Infant, premature, Macronutrientes, Leite humano, Recém-nascido prematuro

## Abstract

**Objective:**

To analyze the composition of macronutrients present in the milk of mothers of preterm newborn infants (PTNB) - protein, fat, carbohydrate, and calories - by gestational age (GA), chronological age (CA) and maternal variables.

**Methods:**

Longitudinal study that analyzed 215 milk samples from the 51 mothers of PTNB admitted in three Neonatal Intensive Care Units of Rio de Janeiro from May/2013-January/2014. Milk collection was performed by pickup pump, on a fixed day of each week until discharge. The spectrophotometric technique with Infrared Analysis (MilkoScan Minor 104) was used for the quantitative analysis. A sample of 7 mL of human milk was taken from the total volume of milk extracted by the mother. The data was grouped by GA (25-27, 28-31, 32-36, 37-40 weeks) and by CA (zero to 4, 5-8, 9-12, 13-16 weeks).

**Results:**

Protein, carbohydrate, fat and calories did not show any pattern of change, with no difference among groups of GA. When the macronutrients were analyzed by groups of CA, protein decreased, with significant difference between the first two groups of CA. Carbohydrates, fat and calories presented increasing values in all groups, without significant differences. Weight gain during pregnancy, maternal hypertension and maternal age were associated with changes in fat and calories in the first moment of the analysis of milk.

**Conclusions:**

There was a significant decrease in the levels of protein during the first eight weeks after birth. CA may be an important factor in the composition of human milk.

## RESUMO

**Objetivo:**

Analisar a composição dos macronutrientes presentes no leite de mães de recém-nascidos pré-termo (RNPT) - gorduras, carboidratos e calorias - por idade gestacional (IG), idade cronológica (IC) e variáveis maternos.

**Métodos:**

Estudo longitudinal para analisar 215 amostras de leite de 51 mães de RNPT admitidos em três unidades neonatais do Rio de Janeiro de maio/2013 a janeiro/2014. A coleta de leite foi realizada por bomba coletora, em dia fixo a cada semana até a alta. Utilizou-se a técnica espectrofotométrica com análise de infravermelho (MilkoScan Minor 104) para a análise quantitativa. Uma amostra de 7 mL de leite humano foi retirada do volume total de leite extraído pela mãe. Os dados foram agrupados por IG (25-27, 28-31, 32-36 e 37-40 semanas) e por IC (0-4, 5-8, 9-12 e 13-16 semanas).

**Resultados:**

Proteínas, carboidratos, gorduras e calorias não apresentaram nenhum padrão de mudança, não havendo diferença entre os grupos de IG. Quando os macronutrientes foram analisados por grupos de IC, a proteína diminuiu, com diferença significante entre os dois primeiros grupos de IC. Carboidratos, gorduras e calorias apresentaram valores crescentes em todos os grupos, sem diferença estatística. O ganho de peso durante a gestação, a presença de hipertensão arterial e a idade materna foram associados a alterações de gordura e calorias no primeiro momento da análise do leite.

**Conclusões:**

Observou-se redução estatisticamente significante nos níveis de proteína durante as primeiras oito semanas após o nascimento. A IC pode ser um fator importante na composição do leite humano.

## INTRODUCTION

Survival rates of preterm newborns (PTNB) have increased substantially over the past two decades.^1^ However, despite advances in nutrition knowledge for this group, postnatal growth restriction continues to be a critical problem.^2^ ^,^ ^3^ ^,^ ^4^ ^,^ ^5^ Providing adequate nutrition in the neonatal period, of which human milk is a part, is a determining factor for immediate survival and for growth and development during childhood, besides being the most important condition for health in the long run.^6^ ^,^ ^7^

According to Arslanouglu et al.,^8^ solid evidence suggests that not only low caloric intake, but also inadequate protein intake is one of the main factors responsible for slow growth and worse neurocognitive results found in PTNBs. The optimization of nutritional support is the best strategy to reduce and/or prevent this restriction.

Several factors can influence the composition of human PTNB milk, which varies greatly, and it is important to analyze the presence of micronutrients to assess the need for supplementation^9^ and energy macronutrients for the necessary adjustments. Bauer and Gerss,^10^ in a recent longitudinal analysis of macronutrients present in the milk of mothers of preterm infants in the first eight weeks of lactation, showed higher concentrations of protein in first weeks, with progressive decrease, in addition to a progressive increase in fat, carbohydrates and calories rate, reaching values higher than in the milk of mothers of term newborns.

Human milk is the best food for term and preterm newborns. However, although the range of scientific studies that discuss the nutritional composition of PTNB breast milk (BM) is wide,^5^ ^,^ ^6^ ^,^ ^7^ ^,^ ^8^ they are still scarce and controversial, especially at the national level, which analyzes the composition of energy macronutrients (carbohydrates, proteins and lipids) present in the mothers’ milk of very low birth weight infants, considering the postnatal chronological age, corrected gestational age and the possible association with some maternal variables, which was the objective of this study.

## METHOD

A mixed longitudinal study was carried out addressing the composition of macronutrients in the milk of mothers of very low birth weight (VLBW) newborns, in the Neonatal Intensive Care Unit (NICU) of *Instituto Fernandes Figueira* (IFF) and at the Perinatal Clinic (units of Barra and Laranjeiras).

All mothers of preterm infants admitted to one of these Neonatal Intensive Care Units (NICUs) who were able to donate sufficient volume of milk for the study were included, as well as mothers of newborns with congenital malformations or with the human immunodeficiency virus (HIV).

To calculate the sample size, we used the study developed by Bauer and Gerss,^10^ which determined the mean difference in protein concentration in the breast milk between the 25th and 32nd weeks of corrected gestational age of the second week of lactation (period of probable start of milk collection). For the sample size, it was established that the differences in means should not exceed 5% of the obtained in the study (32 mothers) for a test power of 80%.

Breast milk collection was performed at the Human Milk Bank by trained nurses, using a collection pump and completely emptying the breast at each collection. The first collection took place when the mother was able to remove enough milk (7 mL) for data analysis. After that start, the milk was collected weekly until hospital discharge.

The quantitative analysis of the energetic macronutrients (proteins, fats and carbohydrates - fundamental food components for the organism) of human milk took place at the Quality Control Laboratory of the IFF’s Milk Bank using the spectrophotometry technique by infrared analysis (MilkoScan Minor 104), already validated for the analysis of human milk.^11^ This method allows the measurement of milk fat, protein and lactose. For this dosage, a 7 mL sample of human milk was needed, which was taken from the total volume of milk collected from the mother.

Milk samples were examined on the same day of collection, with a maximum interval of three hours between collection and analysis. When it was necessary to transport the sample for analysis (milk collected from mothers at the Perinatal Clinic), samples were kept in a thermal box capable of keeping the temperature at 3 to 5ºC, controlled by a specific thermometer attached to it.

The energy macronutrients present in the breast milk were analyzed by age ranges adjusted to the degree of prematurity (GA), by chronological age (CA) and by days of life after birth. For the analysis by GA ranges, data were grouped arbitrarily into four groups, to facilitate the comparison between ages:

- Group 1: 25 to 27 weeks.
- Group 2: 28 to 31 weeks.
- Group 3: 32 to 36 weeks.
- Group 4: 37 to 40 weeks.

For the analysis by CA ranges, data were divided into weeks:

- Group 1: from birth to four weeks.
- Group 2: 5 to 8 weeks.
- Group 3: 9 to 12 weeks.
- Group 4: 13 to 16 weeks.

Macronutrients were also analyzed in relation to the following maternal characteristics: maternal age, pre-gestational body mass index (BMI), weight gain during pregnancy, gestational diabetes mellitus (GDM), systemic arterial hypertension (SAH), maternal infection, twinning, and ethnicity. Mothers were considered to have GDM when their glycemic curve or oral glucose tolerance test were altered, and SAH patients were mothers who already had chronic hypertension before pregnancy, in addition to mothers who presented with pregnancy-specific hypertensive diseases (PSAH), pre-eclampsia and eclampsia. Ethnicity was classified as white, brown or black (self-reported). BMI was calculated by dividing pre-gestational weight into kilos (kg) by height in meters (m) squared and classified as low weight (<18.5), adequate weight (18.5-24.9), overweight (25-29.9) and obesity (>30). Weight gain during pregnancy was based on the recommendations by the Institute of Medicine, taking into account the patient’s pre-gestational BMI, and was classified as deficient, adequate or excessive.

The data collected were recorded in a questionnaire designed for the study and analyzed using the Statistical Package for the Social Sciences (SPSS) version 20. The analysis of variance (ANOVA) was used to test the hypothesis that the concentrations of each macronutrient in the breast milk are equal, on average, according to different GA and CA ranges, using the Levene test to test the homogeneity of data. Additionally, a multiple comparisons test was performed (Tukey and Dunet tests). To assess whether maternal gestational factors determine differences in the macronutrients present in the milk of mothers of preterm infants, the Student’s t test for independent samples and the Mann-Whitney test were performed when the assumption of normality was not verified. The level of significance adopted was 5%.

This study was conducted following good clinical practices and Resolution No. 466/12, being approved by the IFF Research Ethics Committee, Certificate of Presentation for Ethical Appreciation (CAAE) 11414912.0.1001.5269. The free and informed consent form was requested from the mothers of hospitalized newborns and only after their authorization and signature were they included in the study.

## RESULTS

215 milk samples were collected from 51 mothers, from May 2013 to January 2014. All newborns of mothers participating in the study were discharged before 37 weeks and, for this reason, the GA Group 4 (37-40 weeks) was not analyzed. The first milk collection took place on average 19 days after birth.

The analysis of breast milk’s macronutrients showed a progressive increase in carbohydrates when comparing GA groups, while proteins decreases progressively; these differences, however, were not statistically significant when the three GA groups were compared with each other. Total fat and calories did not present any pattern of change (Table 1).

**Table 1:** Concentration of macronutrients in the breast milk of mothers of preterm newborns by gestational age ranges, expressed as mean and standard deviation.

Macronutrient (/100 mL)	25-27 weeks (n=6 lactating mothers)	28-31 weeks (n=20 lactating mothers)	32-36 weeks **(n=25 lactating mothers)**
Fat (g)	3.54±0.95	2.80±1.33	3.37±1.44
Protein (g)	2.28±0.55	1.78±0.98	1.66±0.50
Carbohydrate (g)	6.06±0.30	6.11±0.59	6.28±0.63
Calories (Kcal)	65.24±10.49	55.91±12.06	62.34±11.73

**Figure 1:**
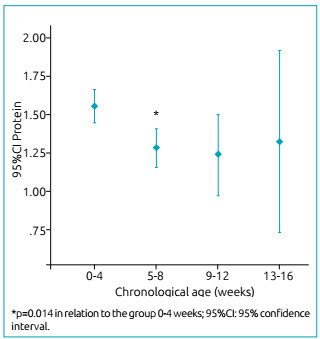
Protein concentration found in the milk of mothers of very low birth weight newborns by groups of chronological age, expressed as mean and standard deviation.

The macronutrients were also analyzed by CA groups. Fat, carbohydrates and calories did not show significant variation, but the protein concentration had statistically significant differences when the first group (zero to four weeks) was compared with the second (five to eight weeks) (p=0.014) (Figure 1).

When analyzing the macronutrients present in the breast milk (in the first moment of analysis) in relation to maternal characteristics, a significantly higher fat and calorie values were found in mothers with adequate weight gain during pregnancy, when compared to mothers with excessive weight gain. The fat and calories values were still statistically lower in the milk of mothers who had SAH during pregnancy as compared to mothers who did not have this condition.

Another variable that showed this same pattern of alteration in the composition of fat and calories was maternal age: mothers aged 35 years or older had a higher concentration of these macronutrients when compared to mothers younger than 35 years (Table 2). The variables weight gain, SAH during pregnancy and maternal age were included in the regression model, and it was found that absence of SAH during pregnancy (p=0.043) and maternal age ≥35 (p<0.001) contributed significantly to increased levels of fat and calories in the milk.

**Table 2:** Association between maternal factors and the concentration of macronutrients in milk at the first moment, expressed as mean and standard deviation.

Maternal factors	Macronutrients (/100 mL)			
	Fat (g)	Protein (g)	Carbohydrate (g)	Calories (Kcal)
BMI				
Low weight (n=2)	4.63±0.47	1.57±0.37	6.50± 0.28	73.89±4.63
Adequate weight (n=29)	3.23±1.20	1.80± 0.91	6.30±0.28	60.94±10.95
Overweight (n=15)	2.73±1.43	1.74±0.42	6.21±0.52	56.87±12.70
Obesity (n=5)	3.49±1.98	1.83±0.60	5.31 ± 1.28	60.01±16.21
Weight gain				
Low (n=26)	3.12±1.29	1.74±0.95	6.33± 0.30	59.67±11.80
Adequate (n=17)	3.75^a^±1.43	1.82±0.50	5.94±0.72	64.82^b^±12.05
Excessive (n=8)	2.09±0.63	1.81±0.33	6.24±0.85	51.85±8.57
GDM				
Yes (n=2)	3.42±1.24	1.26±0.42	6.19±0.06	60.57±13.05
No (n=49)	3.16±1.37	1.80±0.75	6.18± 0.60	60.14±12.14
SAH				
Yes (n=20)	2.63^c^±0.86	1.64 ± 0.45	6.10±0.57	55.07^d^±8.03
No (n=31)	3.51±1.52	1.87±0.88	6.24±0.60	63.44±13.12
Infection				
Yes (n=13)	2.85±1.54	1.58±0.61	6.12±0.90	56.42±12.96
No (n=38)	3.27±1.30	1.85±0.78	6.21±0.45	61.44±11.71
Twinning				
Yes (n=12)	2.74±1.06	1.74±0.35	6.21±0.69	57.05±10.43
No (n=39)	3.30±1.42	1.79±0.83	6.18±0.56	61.12±12.46
Ethnicity				
White (n=37)	3.23±1.20	1.68±0.53	6.31±0.36	61.05±10.54
Brown (n=11)	2.70±1.65	2.07±1.21	5.80±0.96	54.85±14.10
Black (n=3)	4.10±2.06	1.91±0.90	6.02±0.65	68.70±18.66
Age				
<35 (n=32)	2.58±0.97	1.83±0.81	6.22±0.48	23.41±8.77
≥35 (n=19)	4.14^e^±1.37	1.68±0.61	6.11±0.73	37.26^e^±12.34

BMI: body mass index; GDM: gestational diabetes mellitus; SAH: systemic arterial hypertension; ^a^compared to the variable excessive weight gain (p=0.010); ^b^compared to the variable excessive weight gain (p=0.030); ^c^compared to the SAH variable “no” (p=0.012); ^d^compared to the SAH variable “no” (p=0.007); and compared to the variable age <35 (p<0.001)

## DISCUSSION

Human milk is the ideal food for term and preterm newborns, as it adapts nutritionally to the specific needs of infant growth and its use is associated with several benefits for PTNB, both in the short and long run.^12^

With regard to the composition of breast milk, our results are similar to that found by Lafuente et al.^13^ and Bauer and Gerss,^10^ in which the protein followed a pattern of decrease related to corrected age, or weeks of lactation throughout the first weeks; however, the statistical difference between the values of protein by GA reported by these authors was not verified in our study.

An inverse relationship between protein concentration and CA was seen in two studies, suggesting the need for a higher protein content in the first weeks of a preterm newborn’s life, in order to meet the need for a greater daily supply of this macronutrient.^14^ ^,^ ^15^

Ziegler^15^, in a study on the nutritional needs of low birth weight newborns, discusses some nutritional requirements and official recommendations for this group. Among them, the most important recommendation that addresses the role of proteins is from the European Society for Pediatric Gastroenterology Hepatology and Nutrition (ESPGHAN) 2010 (3.5-4.5 g/kg/day).

A 2014 review^16^ found that adequate protein and energy intake, as well as growth rate, is predictive of better long-term health. Recent articles^17^ ^,^ ^18^ raise the hypothesis that the current protein recommendation early in life is not enough to improve outcomes in the growth and development of PTNBs. According to the authors, the greater intake of amino acids by these newborns can increase the speed of weight gain and achieve lean mass compatible with that of term babies.

Carbohydrate showed a progressive increase over CAs, but without statistical difference. This pattern of increase was similar to what Bauer and Gerss found.^10^ Fat and calories data differ in part from what the authors mentioned; in our analysis they did not change per CA. The results achieved suggest that the sample size may be an important limitation of our study, since its calculation was based on the average protein difference. For the study of carbohydrates and fat, the sample size should probably have been larger.

A systematic review and meta-analysis conducted by Gidrenwicz and Fenton in 2014^19^ reported that the protein concentration in breast milk decreases after birth, over six weeks. According to the authors, the difference in protein concentration found between the milk of mothers of full-term and preterm newborns lasts until 3 months of age.

When comparing the mean values of macronutrients found in this study according to CA with data from the literature, the mean values of protein, fat, carbohydrate and calories obtained in the milk samples of mothers of VLWNB in the first four weeks of CA almost reached the values found by Anderson et al.,^20^ who performed this analysis for milk samples from mothers of preterm newborns (28 to 36 weeks) in the first two weeks of CA, however the average values of protein and fat are lower than those identified by Weber et al.^21^ in mothers of LBWNB in the first four weeks of CA.

The study by Weber et al.^21^ also showed that the concentrations of fat present in the milk of mothers of the VLWNB were lower in the morning, a difference not seen for protein values. Kociszewska-Najman et al.^16^ also showed a lower concentration of fat in the milk of mothers of premature infants in the morning. Our samples were collected only in the morning, which could justify the lower values of fat for CA found in our study in relation to those of Weber et al. ^21^, in which milk samples were collected in four periods of the day, and those of Bauer and Gerss^10^, in which samples were collected over a 24-hour period. Another variable that must also be taken into account is the different methods used for the analysis of macronutrients.

The chemical composition of human milk is linked to maternal metabolism, which directly influences its quality and amount.^22^ ^,^ ^23^ Reports correlating body composition, diet and maternal parity affecting human milk macronutrients are not recent.^24^ When analyzing the association between maternal characteristics and macronutrients present in the breast milk in the first analysis, fat and calorie values were higher in mothers with adequate weight gain during pregnancy, with statistical difference when compared to mothers with excessive weight gain. Nutritional status and its association with breast milk macronutrients have been described in the literature, showing a direct relationship between good nutritional status and higher concentrations of fat.^24^ ^,^ ^25^

Some of the studies used maternal BMI for this analysis, which is positively associated with the fat content of milk,^25^ ^,^ ^26^ but they did not take into account weight gain during pregnancy. The effect of weight gain or gestational BMI on the components of breast milk is still controversial. In the joint analysis of data, in a regression model, weight gain during pregnancy was not an impacting factor in the composition of milk in our sample. A limitation of the study is the fact that our methodological design does not control maternal diet during pregnancy, which can be a confounding bias; however, the study by Argov-Argaman et al., published in 2017,^27^ used a food questionnaire and showed no difference in maternal fat consumption, suggesting that the differences in the composition of milk between groups are probably attributed to metabolic and physiological differences, and not to the mother´s diet.

Another maternal variable evaluated in our study was SAH. In Brazil, it is the disease that most often complicates pregnancy, affecting 5 to 10% of pregnant women.^28^ There is no evidence in the literature to support the direct relationship between SAH and the composition of milk, but in our study the value of fat and calories in the milk of mothers who had SAH during pregnancy was lower when compared to mothers who did not have it (p=0.012 for fat and p=0.007 for calories), with no difference in the concentration of carbohydrates or proteins. Controversially, Massmann et al.^29^ evaluated the nutritional composition of milk of women with SAH and found higher levels of total protein in the colostrum and mature milk from hypertensive mothers, but did not analyze fat or calorie levels. No other studies were found associating macronutrients in the breast milk and SAH during pregnancy, but the relationship between this type of disease and maternal age has already been cited in the literature.^30^

Our data indicate a direct relationship between maternal age and the concentration of fat and calories in the milk of mothers over 35 years of age, in comparison with the milk of mothers below that age. This was also discussed by Argov-Argaman et al.^27^ in a study on the relationship between milk composition and maternal age. These authors concluded that the mother’s age is an additional factor that influences the maturation of milk, in terms of fat content and composition, during the later stages of pregnancy. Another study, even more recent, described as the first report on the significant interactions between maternal age and BMI affecting macronutrients in human milk, suggests that both should be considered as impacting factors.^25^

The increase in the age of women at childbirth is a reality that has been growing in recent decades. Late pregnancy, in turn, increases the chances of diseases such as SAH, maternal diabetes and premature birth. Metabolic factors associated with maternal age can affect the composition of milk. However, when assessing significant differences related to SAH between different maternal age groups, we did not observe any association (p=0.554), which leads us to believe that, among the variables analyzed, maternal age was the one with the greatest impact on the composition of breast milk’s macronutrients. These findings reinforce that maternal age must be taken into account when planning diets for pregnant women of different age groups, as well as the importance of adequate prenatal care, when one must encourage breastfeeding, monitor nutritional status, adequate weight gain and blood pressure control. These precautions aim at a better supply of nutrients for the baby, including fat and calories, and thus they can contribute to better nutrition and growth, especially for the VLWNB, who need a higher caloric intake.

Breastfeeding strategies that can have a favorable impact on milk production should be encouraged not only in the admission of a newborn, but also in prenatal care and throughout hospitalization. Support for mothers of preterm infants is essential, aiming to improve and preserve their milk production. This reflects the contemporary trend to support lactation and the awareness of researchers and caregivers about the impact of breast milk on the health of preterm newborns.

According to Victora et al.,^16^ human milk is not only a perfectly adapted food for babies, but probably the most specific personalized medicine that the baby will receive, offered at a time when gene expression is being adjusted for life.
